# Steering Algorithm for a Flexible Microrobot to Enhance Guidewire Control in a Coronary Angioplasty Application

**DOI:** 10.3390/mi9120617

**Published:** 2018-11-23

**Authors:** Ali Kafash Hoshiar, Sungwoong Jeon, Kangho Kim, Seungmin Lee, Jin-young Kim, Hongsoo Choi

**Affiliations:** 1 DGIST-ETH Microrobotics Research Center, DGIST, Daegu 42988, Korea; hoshiar@dgist.ac.kr (A.K.H.); jsw919@dgist.ac.kr (S.J.); ies9357@dgist.ac.kr (K.K.); smlee@dgist.ac.kr (S.L.); jy.kim@dgist.ac.kr (J.-y.K.); 2 Department of Robotics Engineering, DGIST, Daegu 42988, Korea

**Keywords:** flexible microrobot, angioplasty, coronary artery disease, magnetic steering

## Abstract

Magnetically driven microrobots have been widely studied for various biomedical applications in the past decade. An important application of these biomedical microrobots is heart disease treatment. In intravascular treatments, a particular challenge is the submillimeter-sized guidewire steering; this requires a new microrobotic approach. In this study, a flexible microrobot was fabricated by the replica molding method, which consists of three parts: (1) a flexible polydimethylsiloxane (PDMS) body, (2) two permanent magnets, and (3) a micro-spring connector. A mathematical model was developed to describe the relationship between the magnetic field and the deformation. A system identification approach and an algorithm were proposed for steering. The microrobot was fabricated, and the models for steering were experimentally validated under a magnetic field intensity of 15 mT. Limitations to control were identified, and the microrobot was steered in an arbitrary path using the proposed model. Furthermore, the flexible microrobot was steered using the guidewire within a three-dimensional (3D) transparent phantom of the right coronary artery filled with water, to show the potential application in a realistic environment. The flexible microrobot presented here showed promising results for enhancing guidewire steering in percutaneous coronary intervention (PCI).

## 1. Introduction

During the past decade, many applications have been developed for microrobots, from targeted drug and cell delivery [1,2,3,4,5] to minimally invasive surgery [6,7,8,9]. Recently, soft flexible microrobots have been developed for targeted drug delivery [10]. The flexible structure of these microrobots makes them ideal for minimally invasive surgical interventions.

One surgical application in which flexible microrobots can be used is percutaneous coronary intervention (PCI). PCI, also known as angioplasty, is a minimally invasive intervention for treatment of chronic total occlusion (CTO) [11]. During the angioplasty procedure, the surgeon inserts a catheter and guides it toward the coronary artery. The surgeon then manually guides a submillimeter-sized guidewire toward the blood clot. Finally, a secondary catheter follows the guidewire path to open the blood clot and place the stent. The most challenging aspect of this procedure is the micrometer-sized guidewire steering, which relies on the surgeon’s skill. To enhance performance, several robot-assisted technologies have been developed.

An electromechanical navigation system (Magellan robotic system) was developed for catheter steering in angioplasty applications [12,13,14]. The system consists of electromechanical parts for achieving rotational and rectilinear motions of a catheter and an X-ray monitoring system. Despite interesting clinical results, limitations of the system included low control over the tip of the catheter and expensive disposable parts.

A magnetic resonance imaging (MRI)-based magnetic navigation system has been proposed for angioplasty applications. However, the time-consuming image reconstruction limited its application [15,16]. Tailored magnetic steering systems have also been studied for rotational motion during the angioplasty procedure. In the Stereotaxis Niobe magnetic steering system, two permanent magnets and an X-ray monitoring device were used for rotational steering [17]. Although the system showed encouraging results, the mechanical response was slow in terms of changing the field direction, and inability to switch the magnetic field led to the development of electromagnetic navigation systems. Initially, an electromagnetic navigation system with eight coils was proposed for rotational steering of the catheter [18]. Although an X-ray monitoring system was used, the monitoring was not done in real-time. Furthermore, the system had limited access to the patient.

In electromagnetic systems, the input current is controlled. Therefore, in real-time the field direction and magnitude can be changed. The microrobots response to the magnetic field is in real-time and can be controlled in 3D [7]. Furthermore, to improve microrobot control performance, time-delay control was used [19]. The Aeon navigation system was proposed to achieve real-time X-ray monitoring and electromagnetic steering [20]. Despite recent developments in electromagnetic navigation systems, the system should be integrated with a microrobot for challenging sub-millimeter guidewire steering.

Initially, millimeter-sized catheters equipped with permanent magnets were proposed for angioplasty applications, and several nonlinear models were proposed for controlling the steering of these millimeter-sized catheters [21,22,23,24]. Microrobots as 2D or 3D structures in micrometer scale were controlled to perform a task in sub-millimeter. The external energy sources (magnetic, acoustic, etc.) actuate the artificial microrobots [1,2,9]. More recently, a flexible microrobot mounted at the end of a conventional guidewire was introduced [25]. This novel microrobot has a diameter of 500 μm, is steered by the magnetic field, and performs the guidewire steering.

The microrobot is steered using a dedicated actuation system with eight electromagnets. The use of an electromagnetic actuation (EMA) system improves steering efficiency, and the biocompatible design of the microrobots makes the scheme ideal for the future in vivo applications [25]. The proposed scheme, however, had a one-dimensional (1D) steering model. In this paper, we propose a two-dimensional (2D) steering algorithm to further enhance the system performance and enable steering. The proposed algorithm improves the previous 1D steering performance significantly and enables magnetic steering in an arbitrary path.

**Figure 1:**
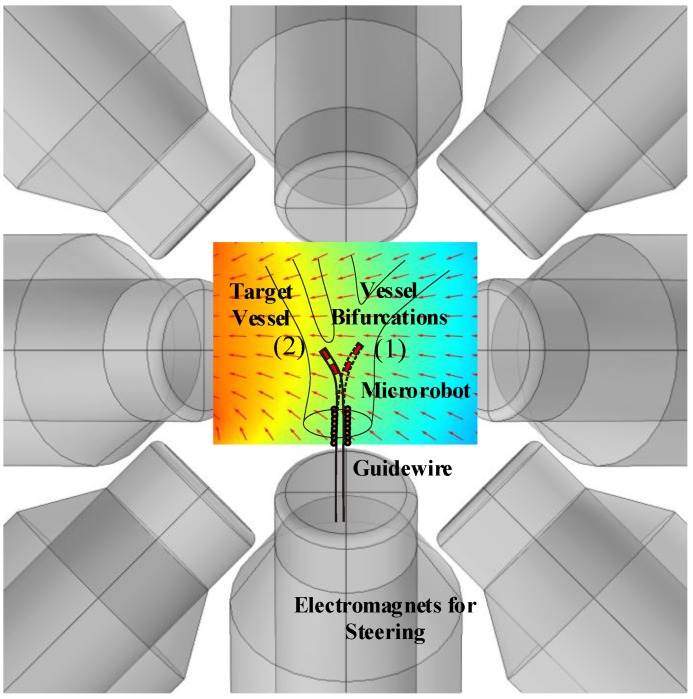
The schematic for flexible microrobot steering.

The schematic of the system is illustrated in Figure 1. Once the microrobot reaches the vessel bifurcation, the magnetic field direction is changed. This change in field direction imposes a torque on the magnets and leads to deformation of the flexible body of the microrobot. This concept is used to steer the microrobot from its initial position (1) to the desired position (2) in Figure 1. After the microrobot was steered using the external magnetic field, the guidewire advances forward manually and follows the microrobot to the desired path. Consequently, the guidewire steering performance is improved by the microrobot.

The final steering system will consist of a tailored X-ray system that can provide top and side views, and will be utilized for real-time monitoring. In this paper, for simplicity, optical cameras are used for monitoring. A modeling approach is utilized to estimate the required inputs for the magnetic field. However, since the steering process is user-supervised, the final position of the microrobot can be manually adjusted. Therefore, a feedforward approach is used in this paper for the modeling.

This paper is organized as follows: in Section 2, the schematic of the proposed microrobotic system is introduced. In Section 3, the magnetic steering model and 2D steering algorithm is developed. In Section 4, the experimental results for 2D and 3D steering are presented. Finally, the conclusion is provided.

## 2. Flexible Microrobotic Platform

**Figure 2:**
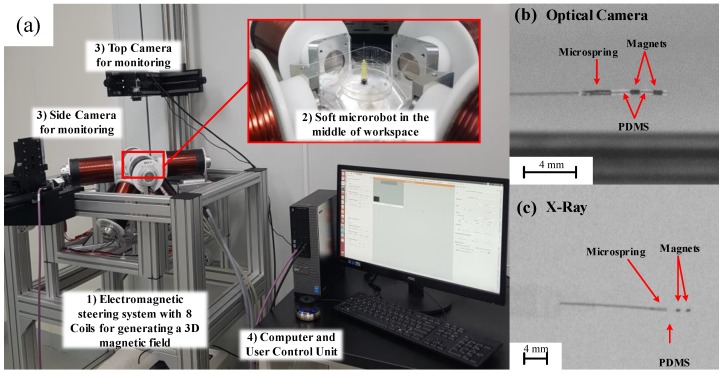
(**a**) The experimental setup for microrobot steering with the OctoMag system, (**b**) Optical image of the microrobot, (**c**) X-ray image of the microrobot.

The magnetic steering system was composed of four parts (Figure 2a): (1) an external EMA system (OctoMag; Aeon Scientific GmbH, Switzerland) that generates a 3D magnetic field (nominal maximum magnetic field intensity is 120 mT, and maximum field for continues use is 40 mT this data provided by the manufacturer, for design details see [7]), (2) a novel flexible microrobot (Figure 2b shows the optical camera view of the microrobot and Figure 2c shows the microrobot in an X-ray image), (3) two optical cameras (side and top views), used for simplicity and which will be replaced by an X-ray system in future studies, and (4) a computer and user interface. Since the ultimate goal is to achieve human scale workspace, achieving acceptable performance in lower magnetic intensity is the desired objective. Therefore, in the previous work, 15 mT was considered for microrobot steering [1,2]. Furthermore, the relationship between different field intensity and the deformation angle previously introduced and high deformation (132.7°) under 15 mT field intensity was achieved [25].

The OctoMag has a homogeneous magnetic field of 80 mm × 80 mm × 60 mm. In the system, the coils configuration was optimized to provide this homogeneous field [7] and the system was successfully used for magnetic steering of microrobots in a number of studies [1,2,25].

Polydimethylsiloxane (PDMS) (Sylgard 184; Dow Corning Corp., Midland, MI, USA), which has a low elastic modulus and a high Poisson ratio, was used for flexible microrobot fabrication. The novel microrobot consisted of two permanent magnets, which are placed at an equal distance within a PDMS matrix and used to steer the guidewire, and a micro spring to connect the guidewire and microrobot. The OctoMag actuation system [7] can generate a 3D magnetic field of constant magnitude that varies in direction. As the field direction changes, the microrobot experiences a magnetic torque, forcing realignment in the direction of the field. Thus, the microrobot can be magnetically steered in the direction of interest (Figure 1).

The material of micro-spring is cold drawn alloyed steel containing carbon, silicon, manganese. The outer diameter, inner diameter, length, and wire thickness are 500 μm, 380 μm, 2000 μm, and 60 μm, respectively.

The OctoMag system shown in Figure 2 afforded five degrees of freedom (DOFs) to untethered microrobots (three position DOFs, two orientation DOFs) [7]. The system can also control the tethered microrobot (with two DOFs $\theta_{r}$ and $\theta_{y}$) by changing the direction of the magnetic field. The microrobot was positioned in the center of the workspace with the orientation is shown in Figure 2. Two cameras were used to obtain top and side views in real time.

### The Microrobot Fabrication

The microrobot exhibits high deformability. The micrometer scale enables the guidewire with a flexible microrobot to be inserted into coronary arteries, and high-level deformability enables guidance over a wide range of branch angles. Considering these design objectives, the microrobot was fabricated from PDMS and incorporated two permanent magnets (NdFeB, N52; Ningbo Zhonghang Magnetic Materials Co., Ltd, Zhejiang, China). The microrobot was cylindrical. The microrobot geometry and material properties are listed in Table 1.

**Figure 3:**
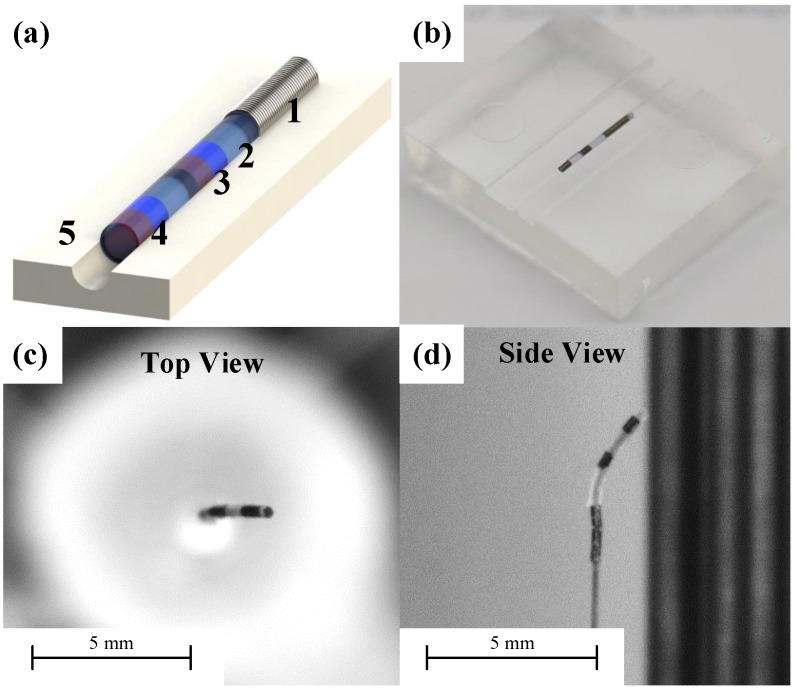
Fabrication of the flexible microrobot. (**a**) The polydimethylsiloxane (PDMS) mold and the microrobot, (1) the micro-spring; (2) PDMS block (3) first magnet (4) second magnet (5) PDMS mold; (**b**) fabricated microrobot on the PDMS mold; (**c**) top, and (**d**) side views of the microrobot during the steering tests.

A replica PDMS mold was prepared as a PDMS master, shown in Figure 3a. As the mold and beam of the microrobot were made of the same material (PDMS), they tended to stick together after the beam was cured. The surface of the PDMS mold was coated with an anti-adhesive layer using the vapor-SAM (self-assembly monolayer) process. First, the surface of the PDMS mold was activated by an oxygen plasma treatment (CUTE; FEMTO SCIENCE, Seoul, Korea). Next, a hydrophobic layer of trichloro (1H,1H,2H,2H-perfluorooclyl) silane (Sigma-Aldrich, St. Louis, MO, USA) was deposited on the PDMS surfaces by a vapor silanization procedure in a vacuum chamber under a pressure of 0.5 bar at 80 °C for 2 h. This surface treatment prevented the beam from adhering to the mold when separated.

First, the PDMS beam (length 1 mm), permanent magnets, and micro-spring were aligned on the mold. The silicone elastomer mixture was then used to fill the mold in the closed chamber under vacuum followed by curing at 80 °C for 8 h. The final structure was deformed under the magnetic field shown in Figure 3c,d. The microrobot could be attached to guidewires of various diameters.

## 3. Modeling for Microrobot Steering

### 3.1. Electromagnetic Force and Torque

Maxwell’s equations define the magnetic field as [25,26]:

(1) $$\nabla \cdot \vec{B}=0$$

(2) $$\nabla \times \vec{B}=\mu_{0}J$$

where B is the 3 × 1 magnetic field vector in the current-free space, $\mu_{0}=4\pi \times 10^{-7}$ $T\cdot A\cdot m^{-1}$ is the permeability of the free space, and *J* is a vector-field describing the electrical current density, which is zero outside the coils. The magnetic force and torque on a magnetic object are given by the following equations:

(3) $$\vec{F}=\left(\vec{m}\cdot \nabla \right)\vec{B}$$

(4) $$\vec{\tau }=\vec{m}\times \vec{B}$$

where $\vec{F}$ and $\vec{\tau }$ are the 3 × 1 force and torque vectors respectively, and $\vec{m}$ is the 3 × 1 dipole moment for the magnetic object. Maxwell’s equations impose two constraints: (1) Equation (1) indicates that the gradient matrix has a zero trace, and (2) Equation (2) shows that the gradient matrix is symmetrical. The force (Equation (3)) is a function of field gradient, whereas the torque is a function of the field (Equation (4)). When steering the microrobot, for simplicity the magnetic gradient is considered to be zero, and magnetic torque (only) is used for steering. Furthermore, the field magnitude is considered to be constant, and the field direction is changed for steering [25,26]. Therefore, the magnetic field is presented as:

(5) $$\begin{array}{c} \left[\begin{array}{c} B_{x}\\ B_{y}\\ B_{z} \end{array}\right]=\left[\begin{array}{ccc} c\;\theta_{y} & -s\;\theta_{y} & 0\\ c\;\theta_{r}s\;\theta_{y} & c\;\theta_{r}c\;\theta_{y} & -s\;\theta_{r}\\ s\;\theta_{r}s\;\theta_{y} & s\;\theta_{r}c\;\theta_{y} & c\;\theta_{r} \end{array}\right]\left[\begin{array}{c} 0\\ B\\ 0 \end{array}\right] \end{array}$$

where *s* and *c* indicate sin and cos, respectively, *r* and *y* are the roll and yaw angles, and *B* is the magnetic field magnitude, which initially is in the *y*-direction. The magnetization matrix in Equation (4) depends on microrobot deformation and is described in the subsequent section.

### 3.2. The Microrobot Deformation

**Figure 4:**
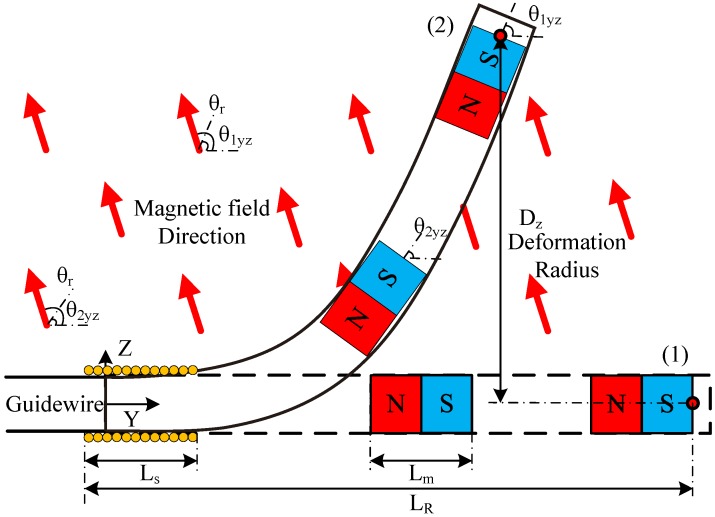
The microrobot steering from the initial position (1) to the desired position (2), deformation in yz-plane under roll angle ($\theta_{r}$) is illustrated, similar deformation is assumed in yx-plane under yaw angle ($\theta_{y}$).

The magnetization of the permanent magnets is initially chosen to be in the Y direction ($\left[\begin{array}{c} 0\;m\;0 \end{array}\right]$). However, matrix M depends on the orientation of the magnets. Figure 4 shows the geometry of the microrobot in the YZ plane, where the orientations of the magnets are represented by $\theta_{iyz}$ (Figure 4) and similarly for the YX plane $\theta_{iyx}$. Therefore, considering both the rotational angles and the directions of initial magnetization, the magnetization vector for each magnet, *i*, can be written as:

(6) $$\begin{array}{c} M=\left[\begin{array}{ccc} 0 & -(s\theta_{iyz}\;c\theta_{iyx}) & c\theta_{iyz}\;c\theta_{iyx}\\ \left(s\theta_{iyz}\;c\theta_{iyx}\right) & 0 & s\theta_{iyx}\\ c\theta_{iyz}\;c\theta_{iyx} & -s\theta_{iyx} & 0 \end{array}\right]m \end{array}$$

Considering Equations (5) and (6), Equation (4) can be written as:

(7) $$\begin{array}{c} \vec{\tau_{i}}=m\;B\;\left[\begin{array}{c} \left(c\theta_{iyz}\;c\theta_{iyx}\right)\left(s\;\theta_{r}c\;\theta_{y}\right)-\left(s\theta_{iyz}\;c\theta_{iyx}\right)\left(c\;\theta_{r}c\;\theta_{y}\right)\\ \left(s\theta_{iyx}s\;\theta_{r}c\;\theta_{y}\right)-\left(s\theta_{iyz}\;c\theta_{iyx}s\;\theta_{y}\right)\\ \left(c\theta_{iyz}\;c\theta_{iyx}s\;\theta_{y}\right)-\left(s\theta_{iyx}c\;\theta_{r}c\;\theta_{y}\right) \end{array}\right] \end{array}$$

where mB shows the magnitude of the torque and the matrix represents the torque direction. Considering the deformation in the YZ plane, Equation (7) is simplified ($\theta_{y}=0$ and $\theta_{iyx}=0$) as:

(8) $$\begin{array}{c} \vec{\tau_{i}}=m\;B\;\left[\begin{array}{c} \sin(\theta_{r}-\theta_{iyz})\\ 0\\ 0 \end{array}\right] \end{array}$$

Considering Equation (8), the equivalent deformation in the Z direction (Figure 4) can be shown as:

(9) $$D_{z}=mB\sum_{i=1}^{n}K_{i}\sin\left(\theta_{r}-\theta_{yzi}\right)$$

where $K_{i}$ is the stiffness and *n* is the number of magnets (in this paper, *n* = 2) in the microrobot. In summary, for a constant magnetic field, the deformation depends on stiffness (mechanical properties), and the difference between the magnet direction $\theta_{yz}$ and the field direction $\theta_{r}$. Therefore, $\theta_{r}$ can be used as an input signal for steering. Similarly, in the YX plane, $\theta_{yx}$ and $\theta_{y}$ determine the deformation and $\theta_{y}$ can be used for the steering.

To achieve higher deformation angles in low magnetic intensity, multi-magnets microrobot is introduced. The number of magnets are defined based on the microrobot geometrical limitation. Since large PDMS structure cannot retain its postures, the microrobot size is confined to 3.8 mm which limits the number of magnets to two. Considering Equation (9) for one and two magnets the ratio of deformation is:

(10) $$\frac{D_{z2}}{D_{z1}}=1+\frac{K_{1}\sin\left(\theta_{r}-\theta_{yz1}\right)}{K_{2}\sin\left(\theta_{r}-\theta_{yz2}\right)}$$

Assuming $\theta_{yz1}$ and $\theta_{yz2}$ are equal, the deformation increase depends on ($K_{1}/K_{2}$) which is always a positive number smaller than 1. This increase was sufficient enough to reach (132.7°) deformation angle under 15 mT field in 1D steering [25].

Furthermore, considering Equation (9), the deformation radius depends on the magnetization magnitude, magnetic field intensity, structural stiffness, and sin of the difference between $\theta_{r}$ and $\theta_{yz}$. Where the angular difference surpasses 90°, the effective force will be reduced. Experimental studies with different field intensities show that for magnetic intensities <15 mT, the angular difference surpasses the threshold (90°) and leads to a decrease in the deformation for higher field directions [25]. Therefore, to avoid this condition and achieve a higher deformation angle, the magnetic intensity was considered to be 15 mT in all experiments. The modeling algorithm to obtain the required $\theta_{r}$ and $\theta_{y}$ for a desired position and orientation of the microrobot end-effector is studied in the subsequent section.

### 3.3. Modeling Algorithms

**Figure 5:**
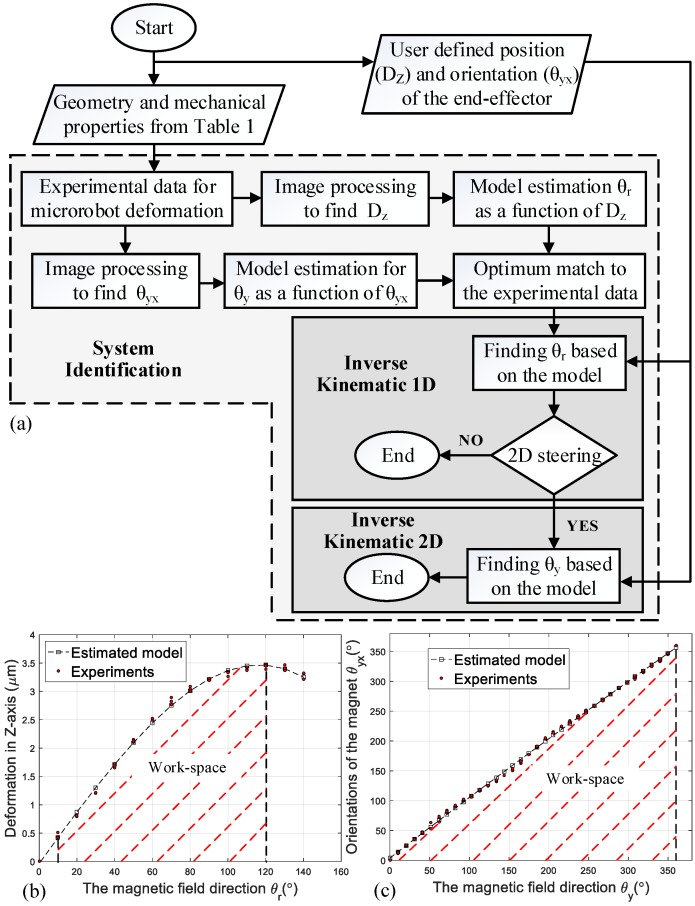
(**a**) The modeling algorithm for one dimensional (1D) and two dimensional (2D) steering; (**b**) the workspace for magnetic field direction $\theta_{r}$, and (**c**) $\theta_{y}$.

Initially, the microrobot’s mechanical properties and geometry were considered using the information in Table 1. The modeling algorithm was divided into three parts: (1) system identification, (2) 1D steering, and (3) 2D steering. Figure 5 shows the flowchart of these three steps. The microrobot was assumed to have isotropic behavior, and the stiffness was assumed to be equal in the *z* and *x* directions.

#### System Identification

It is challenging to analytically model nonlinear deformation in the microrobot. Therefore, in this paper, a system identification approach was used, consisting of three steps: (1) accumulating experimental data, (2) estimating the model for the data, and (3) validating the model.

In the first step, a magnetic intensity of 15 mT was applied, and the magnetic field direction was varied between 0 and 150°. The experiment was repeated three times. The collected data were analyzed using the MATLAB Image Processing Toolbox (MathWorks, Inc., Natick, MA, USA) and the deformation radius ($D_{z}$) and magnetic field direction ($\theta_{r}$) were obtained.

With variation in the field direction, the deformation radius ($D_{z}$) reached its peak and decreased with further elevation in the field direction (Supplementary Video). To simplify the model, the workspace for $\theta_{r}$ was considered to be between 10 and 120°. Thus, the deformation radius will constantly increase with an increase in the field direction $\theta_{r}$.

For the second magnetic field direction ($\theta_{y}$), the field direction was changed between 0 and 360° and the end-effector orientation in the YX plane was obtained. The results for $D_{z}$ with respect to $\theta_{r}$, and $\theta_{yx}$ (end-effector orientation) with respect to $\theta_{y}$ are illustrated in Figure 5b,c respectively.

In the second step, to estimate the system behavior, several models were applied using polynomial and exponential functions. These models were compared with one another and the best match to the experimental data was selected and is shown in Figure 5b,c.

In the third step, to validate the model, within the workspace limits, the $D_{z}$ and $\theta_{yx}$ were changed and maximum deviations between the experimental results and the model for $\theta_{r}$ and $\theta_{y}$ were 7.3% and 11.15%, respectively.

## 4. Results and Discussion

### 4.1. Two Dimensional Steering Experiment

**Figure 6:**
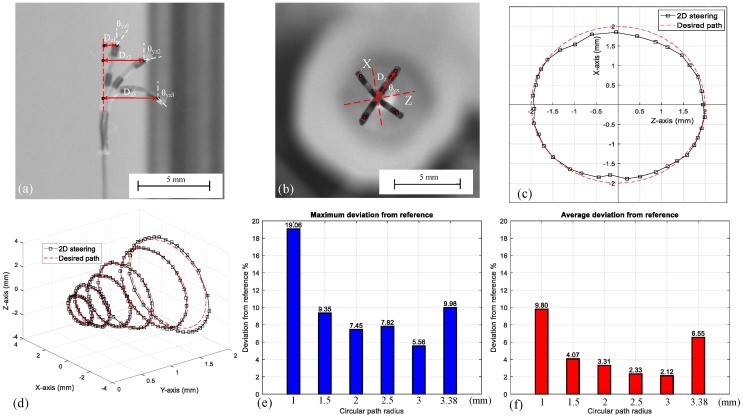
(**a**) 1D steering of a microrobot with different deformation radii ($D_{z}$); (**b**) 2D steering of a flexible microrobot with different deformation directions of the end-effector ($\theta_{yx}$); (**c**,**d**) the compression between the desired path and experimental results ($\theta_{r}$ is 33° and $\theta_{y}$ varied between 0° and 360°); (**e**) 2D steering for different deformation radii $D_{z}$, for the circles (from 1 to 3.38 mm radius) $\theta_{r}$ is 15°, 23°, 33°, 46°, 65°, 105°, respectively, and $\theta_{y}$ is varied between 0° to 360°, (**e**) maximum deviation between the experimental and the desired position (**f**) average deviation between the experimental and the desired position.

Utilizing the flowchart in Figure 5a, and using the model in Figure 5b for any desired deformation radius, the magnetic field direction can be determined. Figure 6a shows the deformation radius under three different field directions ($\theta_{r}$).

To illustrate the 2D steering performance, initially for the desired deformation radius ($D_{z}$), the $\theta_{r}$ was calculated using Figure 5b. Then, to change the orientation ($\theta_{yx}$), the $\theta_{y}$ was varied between 0 and 360°, Figure 6b,c illustrate the change in the end-effector position in 2D steering. A maximum variation of 7.45 % between the experimental results and the desired radius was observed.

Finally, to show the 3D workspace, the deformation radius was varied between 1 and 3.38 mm and for each radius, and $\theta_{y}$ was varied between 0 and 360° (the experimental and desired results are presented in Figure 6d).

Bar charts in Figure 6e,f show the deviation between the desired and experimental path in 2D steering. A maximum deviation of 19.06% in a 1 mm radius was observed. For all other conditions, the maximum deviation remained under 10%. The general trend for all conditions except the 3.86 mm radius was descending. It has been observed that for radius 3.38 mm, the deformation was highly nonlinear, which decreases the reliability. To have a more acceptable agreement between the model and the experiments, the deformation radius was considered to be between 1 and 3 mm. Average coronary artery radius is 1.5 to 2 mm [27]. The upper limit is 3 mm which is bigger than the average coronary artery radius. Although, it is possible to steer the microrobot in smaller radius (<1 mm); to reach higher performance the lower bound is limited to 1 mm.

### 4.2. Microrobot Steering in an Arbitrary Path

**Figure 7:**
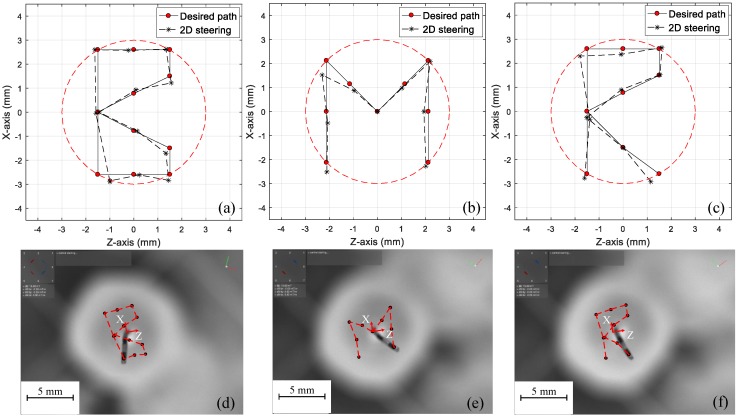
(**a**–**c**) compression between the desired path (using the initial letters of the biomedical micro robot (BMR)) and the experimental results, (**d**–**f**), the experimental results (the red dots are the end-effector position in each step).

To show the designed microrobot performance in an arbitrary path, the initial letters of biomedical micro robots (BMR) were considered. The maximum deformation radius was considered to be 3 mm, and within this limit, the adequate number of points were designated for each letter (shown in Figure 7a–c). The algorithm in Figure 5 was used to find the roll and yaw angles for each point.

**Figure 8:**
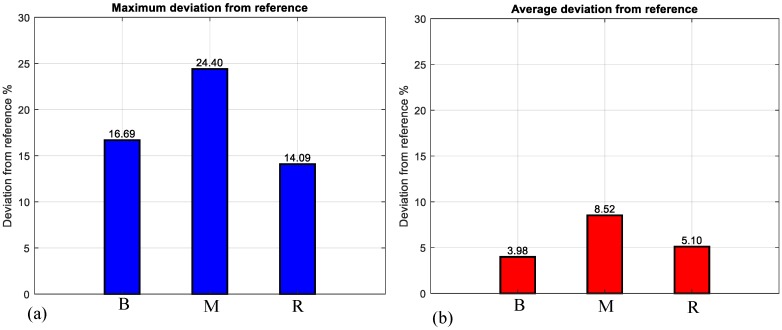
(**a**) Maximum and (**b**) average deviation between the desired path (using the initial letters of the BMR) and the experimental results.

The magnetic field directions ($\theta_{r}$ and $\theta_{y}$) were changed based on the model. Each point position is illustrated by a red dot in Figure 7d–e. Considering the magnetic field direction in the workspace, the position of the end-effector was calculated by image processing and is shown in Figure 7a–c. An acceptable agreement between the desired path and the experimentally obtained results can be observed in Figure 8. For the letters B, M, R maximum deviations of 16.69%, 24.40%, 14.09% were observed, respectively. Table 2 shows the magnetic field directions ($\theta_{r}$ and $\theta_{y}$) for the arbitrary path in Figure 7. The magnetic field intensity was constant (15 mT) in all experiments.

### 4.3. The Guidewire-Steered Microrobot

To show the practical application of the developed microrobot, a transparent 3D phantom of the right coronary artery was filled with water (Supplementary Video). The microrobot steered in the bifurcation magnetically and moved forward in the desired outlet manually. The microrobot was steered using the guidewire in a constant magnetic field of 15 mT magnitude, and only the field direction was changed for steering.

To target the right branch, deformations of 30° (roll), and 330° (yaw) angles were applied. The guidewire was then advanced by hand to pass through the bifurcation point. The guidewire-steered microrobot was pulled back manually to the bifurcation point. To align the guidewire with the microrobot to the left outlet, the field direction was changed to 0° (roll), and 30° (yaw). Then it was advanced manually to enter the outlet. To sum up, the guidewire-steered microrobot exhibited dexterous 3D movement in a 3D path, and could be steered by changing the field direction. The process is illustrated in the Supplementary Video.

## 5. Conclusions

A microrobot was developed to improve intravascular guidewire steerability. The microrobot has a flexible structure and permanent magnets. The external magnetic field was used for steering the microrobot. The deformation angle depended on the magnetic gradient, and the direction and intensity of the magnetic field. The gradient was considered zero, the intensity was held constant (15 mT for all experiments), and the field direction was used for steering. The roll ($\theta_{r}$ varied between 0 and 120°) and yaw ($\theta_{y}$ varied between 0 and 360°) angles are used for steering. An algorithm with the system identification approach was utilized for modeling to enhance microrobot control.

To test a complex arbitrary path, the initial letters of BMR were considered. The microrobot was successfully steered to the desired points to show these letters. For 2D arbitrary path steering with letters B, M, and R maximum deviations of 16.69%, 24.40%, and 14.09% respectively were measured using image processing tools. The practical application of the microrobot steered with a guidewire in a 3D phantom is shown in the Supplementary Video. Thus, our novel microrobot improves guidewire steerability and will find applications in robot-assisted PCI procedures.

The tailored electromagnetic actuation systems show the potentials of the presented scheme for future human scale experiments. Future works will contain the development of an electromagnetic actuation system with two X-rays for a real-time 3D monitoring. To upscale the electromagnetic actuation system, a cooling system should be integrated with electromagnets to enable higher current utilization. Moreover, bigger electromagnetic coils can be used to increase the workspace. Mathematical and FEM models should be developed to optimize the system design.

The English in this document has been checked by at least two professional editors, both native speakers of English. For a certificate, please see: http://www.textcheck.com/certificate/XUT43b.

## Figures and Tables

**Table 1:** Geometrical information and mechanical properties of the microrobot.

	Geometrical Information				Mechanical Properties	
	Diameter (μm)		Length (μm)		Density (kg/m^3^)	Young Modulus (Pa)
Microrobot	$D_{R}$	500	$L_{R}$	3800	0.97 *	$600\times 10^{3}$ *
Magnet	$d_{m}$	400	$L_{m}$	800	7500	$160\times 10^{9}$
Micro-spring	$d_{s}$	500	$L_{s}$	2000	-	-
Guidewire	$d_{g}$	360	$L_{g}$	$1.9\times 10^{6}$	-	-

* PDMS part of the microrobot.

**Table 2:** The magnetic field direction for 2D steering of the microrobot in an arbitrary path.

Point Number		1	2	3	4	5	6	7	8	9	10	11	12	13
Field direction	$\theta_{r}$	65°	23°	65°	48.5°	65°	36°	10°	23°	10°	36°	65°	47°	65°
for letter “B”	$\theta_{y}$	254°	197°	136°	104°	71°	54°	104°	197°	281°	320°	307°	281°	254°
Field direction	$\theta_{r}$	65°	36°	65°	25.5°	0°	25.5°	65°	36°	65°	-	-	-	-
for letter “M”	$\theta_{y}$	254°	197°	152°	152°	2°	54°	54°	2°	320°	-	-	-	-
Field direction	$\theta_{r}$	65°	23°	65°	48.5°	65°	36°	10°	23°	10°	65°	-	-	-
for letter “R”	$\theta_{y}$	254°	197°	136°	104°	71°	54°	104°	197°	281°	307°	-	-	-

## Supplementary Materials

The following are available online at http://www.mdpi.com/2072-666X/9/12/617/s1, Video S1. Deformation Radius Under 15 mT Field Intensity, Two Dimensional Steering, Soft Microrobot Steering in an Arbitrary Path, The Guidewire with Soft Microrobot Steering.

## References

[1] Lee S., Kim S., Kim S., Kim J.Y., Moon C., Nelson B.J., Choi H. (2018). A Capsule-Type Microrobot with Pick-and-Drop Motion for Targeted Drug and Cell Delivery. Adv. Healthc. Mater..

[2] Kim S., Qiu F., Kim S., Ghanbari A., Moon C., Zhang L., Nelson B.J., Choi H. (2013). Fabrication and characterization of magnetic microrobots for three-dimensional cell culture and targeted transportation. Adv. Mater..

[3] Hoshiar A.K., Le T.A., Amin F.U., Kim M.O., Yoon J. (2017). Studies of aggregated nanoparticles steering during magnetic-guided drug delivery in the blood vessels. J. Magn. Magn. Mater..

[4] Amin F.U., Hoshiar A.K., Do T.D., Noh Y., Shah S.A., Khan M.S., Yoon J., Kim M.O. (2017). Osmotin-loaded magnetic nanoparticles with electromagnetic guidance for the treatment of Alzheimer’s disease. Nanoscale.

[5] Hoshiar A.K., Le T.A., Amin F.U., Kim M.O., Yoon J. (2017). A Novel Magnetic Actuation Scheme to Disaggregate Nanoparticles and Enhance Passage across the Blood–Brain Barrier. Nanomaterials.

[6] Lee S., Lee S., Kim S., Yoon C.H., Park H.J., Kim J.y., Choi H. (2018). Fabrication and Characterization of a Magnetic Drilling Actuator for Navigation in a Three-dimensional Phantom Vascular Network. Sci. Rep..

[7] Kummer M.P., Abbott J.J., Kratochvil B.E., Borer R., Sengul A., Nelson B.J. (2010). OctoMag: An electromagnetic system for 5-DOF wireless micromanipulation. IEEE Trans. Robot..

[8] Ullrich F., Bergeles C., Pokki J., Ergeneman O., Erni S., Chatzipirpiridis G., Pané S., Framme C., Nelson B.J. (2013). Mobility experiments with microrobots for minimally invasive intraocular surgery. Investig. Ophthalmol. Vis. Sci..

[9] Nelson B.J., Kaliakatsos I.K., Abbott J.J. (2010). Microrobots for minimally invasive medicine. Annu. Rev. Biomed. Eng..

[10] Charreyron S.L., Zeydan B., Nelson B.J. Shared control of a magnetic microcatheter for vitreoretinal targeted drug delivery. Proceedings of the 2017 IEEE International Conference on Robotics and Automation (ICRA).

[11] Jeong S., Choi H., Go G., Lee C., Lim K.S., Sim D.S., Jeong M.H., Ko S.Y., Park J.O., Park S. (2016). Penetration of an artificial arterial thromboembolism in a live animal using an intravascular therapeutic microrobot system. Med. Eng. Phys..

[12] Riga C.V., Bicknell C.D., Rolls A., Cheshire N.J., Hamady M.S. (2013). Robot-assisted Fenestrated Endovascular Aneurysm Repair (FEVAR) Using the Magellan System. J. Vasc. Int. Radiol..

[13] Thaveau F., Nicolini P., Lucereau B., Georg Y., Lejay A., Chakfe N. (2015). Associated Da Vinci and magellan robotic systems for successful treatment of nutcracker syndrome. J. Laparoendosc. Adv. Surg. Tech..

[14] Wolujewicz M. (2016). Robotic-Assisted Endovascular Pulmonary Artery Foreign Body Retrieval: A Case Report. Vasc. Endovasc. Surg..

[15] Muller L., Saeed M., Wilson M.W., Hetts S.W. (2012). Remote control catheter navigation: Options for guidance under MRI. J. Cardiovasc. Magn. Resonance.

[16] Settecase F., Sussman M.S., Wilson M.W., Hetts S., Arenson R.L., Malba V., Bernhardt A.F., Kucharczyk W., Roberts T.P. (2007). Magnetically-assisted remote control (MARC) steering of endovascular catheters for interventional MRI: A model for deflection and design implications. Med. Phys..

[17] Carpi F., Pappone C. (2009). Stereotaxis Niobe^®^ magnetic navigation system for endocardial catheter ablation and gastrointestinal capsule endoscopy. Expert Rev. Med. Devices.

[18] Filgueiras-Rama D., Estrada A., Shachar J., Castrejón S., Doiny D., Ortega M., Gang E., Merino J.L. (2013). Remote magnetic navigation for accurate, real-time catheter positioning and ablation in cardiac electrophysiology procedures. J. Vis. Exp. JoVE.

[19] Ghanbari A., Chang P.H., Nelson B.J., Choi H. (2014). Magnetic actuation of a cylindrical microrobot using time-delay-estimation closed-loop control: Modeling and experiments. Smart Mater. Struct..

[20] Chautems C., Nelson B.J. The tethered magnet: Force and 5-DOF pose control for cardiac ablation. Proceedings of the 2017 IEEE International Conference on Robotics and Automation (ICRA).

[21] Tunay I. Modeling magnetic catheters in external fields. Proceedings of the 26th Annual International Conference of the IEEE Engineering in Medicine and Biology Society.

[22] Tunay I. (2013). Spatial Continuum Models of Rods Undergoing Large Deformation and Inflation. IEEE Trans. Robot..

[23] Kratchman L.B., Bruns T.L., Abbott J.J., Webster R.J. (2017). Guiding Elastic Rods With a Robot-Manipulated Magnet for Medical Applications. IEEE Trans. Robot..

[24] Edelmann J., Petruska A.J., Nelson B.J. (2017). Magnetic control of continuum devices. Int. J. Robot. Res..

[25] Jeon S., Hoshiar A.K., Kim K., Lee S., Kim E., Lee S., Kim J.y., Nelson B.J., Cha H.J., Yi B.J., Choi H. (2018). A Magnetically Controlled Soft Microrobot Steering a Guidewire in a Three-dimensional Phantom Vascular Network. Soft Robot..

[26] Petruska A.J., Nelson B.J. (2015). Minimum bounds on the number of electromagnets required for remote magnetic manipulation. IEEE Trans. Robot..

[27] Montorsi P., Ravagnani P.M., Galli S., Rotatori F., Briganti A., Salonia A., Rigatti P., Montorsi F. (2005). The artery size hypothesis: A macrovascular link between erectile dysfunction and coronary artery disease. Am. J. Cardiol..

